# Trends on Prevalence, All-Cause Mortality, and Survival Status of Dementia Patients in Rural China Based on Pooling Analysis

**DOI:** 10.3389/ijph.2024.1606786

**Published:** 2024-08-22

**Authors:** Kang Huo, Suhang Shang, Jin Wang, Chen Chen, Liangjun Dang, Ling Gao, Shan Wei, Lingxia Zeng, Qiumin Qu

**Affiliations:** ^1^ Department of Neurology, First Affiliated Hospital of Xi’an Jiaotong University, Xi’an, China; ^2^ Department of Epidemiology and Biostatistics, School of Public Health, Xi’an Jiaotong University, Xi’an, China; ^3^ Center for Brain Science, First Affiliated Hospital of Xi’an Jiaotong University, Xi’an, China

**Keywords:** dementia, prevalence, mortality, survival status, cohort study

## Abstract

**Objectives:**

No study has reported secular trends in dementia prevalence, all-cause mortality, and survival status in rural China.

**Methods:**

We established two cohorts (XRRCC1 and XRRCC2) in the same region of China, 17 years apart, to compare dementia prevalence, all-cause mortality, and survival status, and performed regression analysis to identify associated factors.

**Results:**

Dementia prevalence was 3.49% in XRRCC1 and 4.25% in XRRCC2, with XRRCC2 showing a significantly higher prevalence (OR = 1.79, 95%CI: 1.2–2.65). All-cause mortality rates for dementia patients were 62.0% in XRRCC1 and 35.7% in XRRCC2. Mortality in the normal population of XRRCC2 decreased by 66% compared to XRRCC1, mainly due to improved survival rates in women with dementia. Dementia prevalence was positively associated with age >65, spouse-absent status, and stroke, and negatively associated with ≥6 years of education.

**Conclusion:**

Dementia prevalence in rural China increased over 17 years, while mortality decreased. Major risk factors include aging, no spouse, and stroke, with higher education offering some protection.

## Introduction

China is facing a significant problem of an aging population. According to the UN Department of Economic and Social Affairs, the number of people aged 60 years and older will reach 2.1 billion by 2050, inevitably increasing the number of people with dementia. This number is expected to increase dramatically from 9.5 million in 2020 to 17.8 million in 2050 [1]. In China, the overall prevalence of dementia among people aged 60 years and older is projected to increase from 5.8% in 2020 to 6.7% in 2030 [2], posing significant challenges for families and the national healthcare and social systems [3]. According to the 7th China Census in 2020, over 36% of China’s population lives in rural areas, where dementia is more prevalent (7.2%) compared to that in urban areas (4.8%) [4]. Because of the economic inequity between the urban and rural regions of the country, rural residents in China have limited access to quality healthcare and social support, making them more vulnerable to dementia [5]. Hence, it is necessary to understand the secular trends of dementia to develop a suitable public health policy and set resource priorities to reduce the burden. However, very few studies have reported on the epidemiological trends of dementia, focusing specifically on the rural Chinese population.

Secular trends regarding the prevalence of age-related disorders change for various reasons, including a longer life expectancy for those with chronic diseases, an increase in the number of new cases, patient migration, and improved diagnostic criteria and awareness [6]. Since 2000, the Chinese government has invested billions of dollars to improve rural healthcare to address health inequities with urban areas. Improved insurance coverage has made it easier for rural residents to access essential healthcare services, leading to reduced mortality rates among the rural elderly. This increased life expectancy is more noticeable among people older than 60 years, exacerbating the country’s aging problem and increasing the number of people living with dementia. Therefore, it is crucial to understand the correlation between changes in mortality rates and temporal trends of dementia.

The risk and protection factors of dementia have undergone significant changes in recent years, reflecting the evolving understanding of dementia over the past decades. China’s rapid economic development has Westernized its lifestyle and diet, resulting in a shift in the pattern of prevalent diseases, including an increase in chronic non-communicable diseases such as hypertension [7], stroke [8], diabetes [9], and heart disease [10] and a decrease in infectious diseases. Besides, the country’s fast urbanization over the past two decades has fueled the rapid migration of its rural youth to urban areas, leaving the older people behind and accelerating the aging problem in rural districts, further increasing the risk of cognitive decline and dementia [11]. Individuals from later cohorts tend to have a higher cognitive reserve because of the greater awareness of the importance of education and increased government investment in the education sector. It is crucial to understand how changes in demographic characteristics in China’s rural districts have impacted the shift of risk and protection factors for dementia over time, affecting the secular trends of dementia.

The prevalence of dementia in China varies geographically and ranges from 4.8% to 7.2% [12], with lower rates in Central and Southern China, Hong Kong, and Taiwan and higher rates in Western China [13]. Because of the heterogeneity of the investigational design, diagnostic criteria, sampling methods, and inclusion of the study population, it is challenging to identify secular trends in the prevalence of dementia in China. Therefore, a good understanding of temporal trends in the epidemiology of dementia with representative samples and a stable methodology is needed to develop proactive public health policies for the early identification of high-risk patients and reduce the financial burden on the individual and society. We conducted our investigation using two longitudinal population-based cohorts with the same investigation methods in China, 17 years apart, to investigate temporal trends about the prevalence, mortality, and survival status of patients with dementia and the possible reasons through pooling analysis.

## Methods

### Study Population, Cohort Design, and Ethical Approval

We established two cohorts in the same region of China, which we studied for over 17 years. The first cohort was investigated from September 1997 through December 1998 and was named Xi’an rural resident cognitive cohort 1 (XRRCC1). We used a stratified, multistage cluster-sampling methodology to select the study population with the following inclusion criteria: (1) permanent residents living in villages for more than 3 years; (2) those aged ≥55 years; and (3) those who voluntarily signed the informed consent form to participate. Details of the sampling strategy have been reported previously [14]. Participants who completed the baseline investigation were then followed up from November 2000 through April 2002, with a median follow-up time of 48 months. The details of the follow-up have been described elsewhere [15].

The second cohort study, referred to as XRRCC2, was conducted from October 2014 through March 2015. The same selection method, inclusion criteria, and geographic area (Xi’an) were used as those for the XRRCC1 cohort. Participants who completed the baseline investigation were then followed up in May 2019, with a consistent median follow-up time of 48 months.

The study protocol was approved by the Medical Ethics Committee of the First Affiliated Hospital of Xi’an Jiaotong University (Ethical Approval Number: XJTU1AF2021LSK-038). Voluntary written informed consent was obtained. Proxy respondents were used when the target person had hearing or vision problems.

Xi’an, located in central China, has a population of 12 million, with 25% residing in rural areas. It is a representative investigation sample of the overall Chinese population. The population-distribution characteristics of the two cohorts and the Xi’an rural population corresponding to the study period are listed in the Supplementary Data.

### Interviews, Data Collection, and Follow-Ups

We used standardized protocols and questionnaires to collect sociodemographic information, medical histories, and the survival status of the participants in the two cohorts. The entire investigation comprised baseline face-to-face and follow-up interviews. The interviews were conducted by neurologists and trained medical students. All staff received at least 1 week of concentrated training from supervisors with experience in conducting nationwide cohort studies to correctly use unified questionnaires and standardized survey terms for assessing cognition reserve before community practice. The respondents who failed to meet three times were marked as lost to follow-up. Consistency between the examiners was evaluated afterward using a Kappa coefficient value of 0.76. We used death certificates from the local bureau of statistics to ascertain the vital status of the participants. The enrollees were followed over time, and each death was documented. The time from the start of follow-up to the occurrence of the outcome (death or lost to follow-up) was recorded for each participant. All these time periods were then summed up to calculate the total person-years.

### Definition of Characteristics

All participants were stratified by age. They were also divided based on their level of education (whether they completed more or less than 6 years of school-age education). We placed all single, divorced, and widowed participants in one group named “spouse-absent state.” Hypertension is defined as a systolic blood pressure of ≥140 mm Hg or a diastolic blood pressure of ≥90 mm Hg. Participants who used the antihypertensive therapy even once were also deemed to have hypertension. Under cardiovascular diseases, we placed coronary atherosclerotic heart diseases, atrial fibrillation, and heart failure. Under stroke, we included ischemic or hemorrhage stroke. All the information was collected through the medical records of the participants.

### Diagnosis of Dementia

We used the same three-phase assessment to diagnose dementia in both cohorts. Firstly, a face-to-face interview of all subjects was conducted to obtain information about their demography and medical history. In addition, the Chinese version of the Mini-Mental State Examination (C-MMSE) was applied. Second, participants with a C-MMSE score less than the cutoff points [16] and those who could not complete the screening were further interviewed, and their detailed medical records and data on standard neuropsychological tests were obtained. The preliminary diagnosis was made following the diagnosis criteria of DSM-IV for dementia [17] by an experienced neurologist, attending or above, based on the interviews. Depression or other psychiatric disorders were ruled out. Third, the initial diagnosis was confirmed by repeating face-to-face interviews and examinations 6 months later for the traced person.

### Statistical Analysis

All demographic characteristics at baseline were reported as numbers or percentages. We used the chi-square test for binary variables (age stratification, gender, education, marriage status, and comorbidities) and the Student’s *t*-test or Kruskal–Wallis test for continuous variables to compare differences between the cohorts (dementia or no-dementia groups). We used a multiple-imputation model containing age, gender, and disease status to handle missing data [18]. In total, 15 input datasets were merged into a pooling observation dataset. Then, Rubin’s combination rules were used to form a sum observation dataset for regression analysis.

We directly estimated the crude prevalence and mortality with the number of cases (dementia or death) and the number of participants at the beginning of the survey with a Poisson regression model. We then standardized the prevalence and mortality using the combined population from the two surveys as the standard population and used the age- and sex-distribution ratio of the rural population as the reference. Age-stratified prevalence of dementia in both men and women was obtained and compared using a chi-square test. All rates were shown as percentages with 95% confidence intervals (CIs).

Temporal trends between the two cohorts were analyzed with pooled data using multivariate regression models adjusted for age, sex, and education. These trends were demonstrated in terms of odds ratios (ORs) obtained from logistic regression for prevalence and as hazard ratios (HRs) determined through Cox regression for mortality, using XRRCC1 as the reference. We employed a competing risk model to compare trends between the two surveys by considering hypertension, heart disease, and stroke as the competing factors for dementia.

Kaplan–Meier curves were used to estimate the survival status using the date of the screening interview as the starting point and the date of death or the end of the follow-up as the endpoint. We used the log-rank test to compare differences in the distribution of the survival time of the participants with and without dementia. The factors associated with the changing trends of prevalence in dementia were evaluated using the pooled data of the two cohorts with a fully adjusted multivariate model and shown using a forest plot. Factors with OR values >1 were considered responsible for changing the temporal trends in prevalence between the two cohorts.

We used two additional methods to adjust the missing data to verify whether results differed after using the multiple-imputation method as a sensitivity analysis test; the missing values were assigned the (1) worst-case and (2) best-case scenarios. All statistical analyses were conducted using StataSE16 (StataCorp LP, TX, USA). *P*-values <0.05 were considered statistically significant.

## Results

### Baseline Characteristics and Follow-Up Results of the Two Cohorts

**FIGURE 1:**
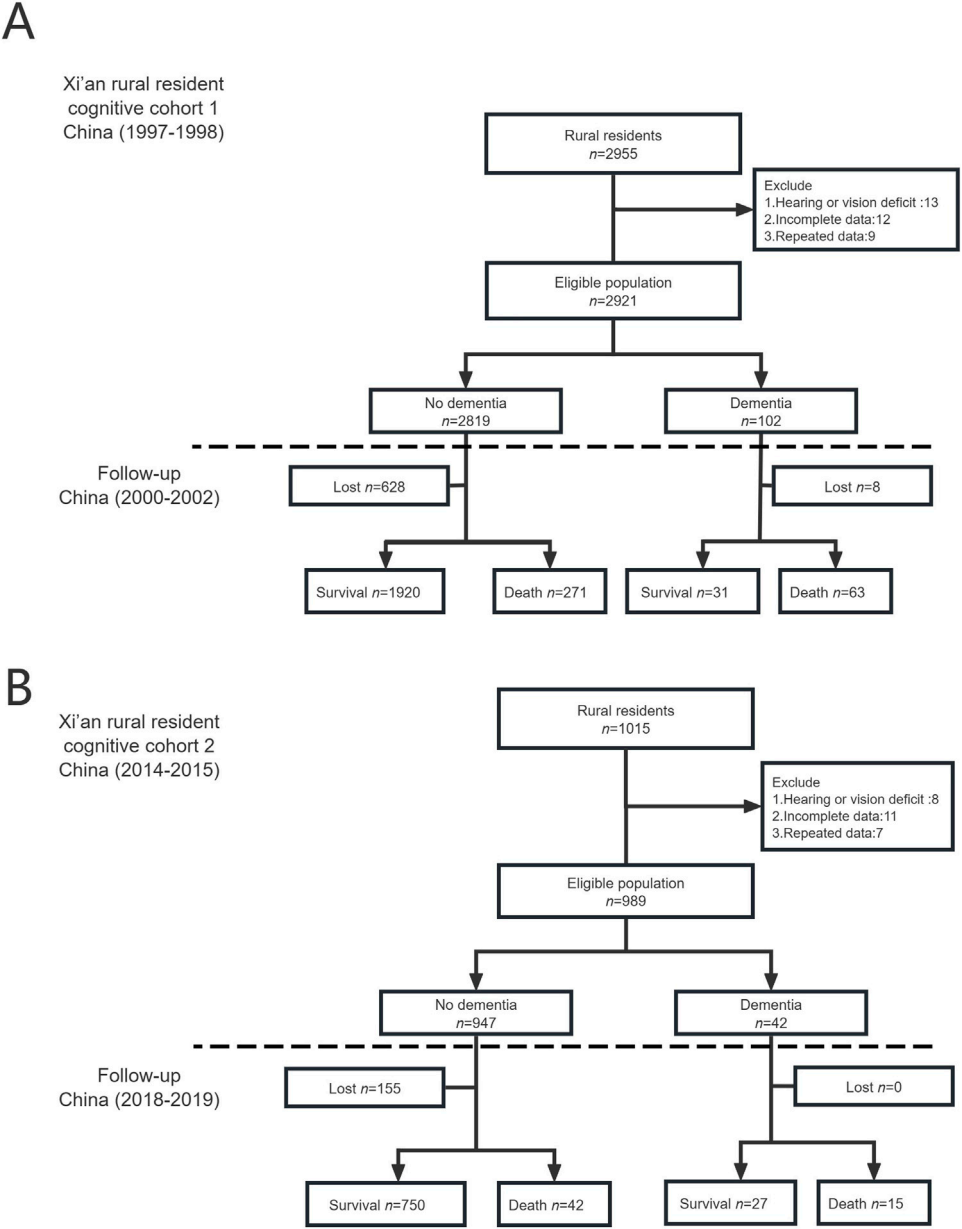
Flow chart of two cohorts (Xi’an rural district, China. 2020). **(A)** The flowchart of Xi’an rural resident cognitive cohort 1. **(B)** The flowchart of Xi’an rural resident cognitive cohort 2.

For the XRRCC1, 2,955 rural residents were interviewed, 2,921 of whom completed the baseline investigation (Figure 1). For the XRRCC2, 1,015 rural residents were interviewed, 989 of whom completed the baseline investigation. The baseline demographics of the participants of both cohorts are listed in Table 1. The gender and age distribution had no significant differences between the two cohorts. Compared with the XRRCC1, the XRRCC2 were more likely to have completed school education. The XRRCC2 had a higher number of married participants and fewer with the spouse-absent status than those in the XRRCC1. The XRRCC2 had a significantly higher prevalence of hypertension (*P* < 0.001). There was no difference in the prevalence of cardiovascular diseases between the two cohorts. The prevalence of stroke did not differ between the two cohorts in the overall population. However, the rate was significantly elevated among participants with concomitant dementia (*P* < 0.001). The overall C-MMSE scores were not significantly different between the XRRCC1 and XRRCC2 participants; however, the mean C-MMSE score was higher in the XRRCC1 than it was in the XRRCC2 participants with dementia (15.12 vs. 13.57, *P* < 0.001).

**TABLE 1:** Demographic characteristics of two cohorts at baseline (Xi’an rural district, China. 2020).

Variables	XRRCC1			XRRCC2			P-value^a^		
	Dementia	No dementia	Total	Dementia	No dementia	Total	P1	P2	P3
No. (*n*, %)	102 (3.49)	2,819 (96.51)	2,921	42 (4.25)	947 (95.75)	989			
Sex (*n*, %)							0.77	0.49	0.54
Men	44 (43.14)	1,179 (41.82)	1,223 (41.87)	17 (40.48)	408 (43.08)	425 (42.97)			
Women	58 (56.86)	1,640 (58.18)	1,698 (58.13)	25 (59.52)	539 (56.92)	564 (57.03)			
Age, y (*n*, %)							0.89	0.1	0.13
55–59	4 (3.92)	650 (23.06)	654 (22.39)	2 (4.76)	290 (30.62)	292 (29.52)			
60–64	7 (6.86)	714 (25.33)	721 (24.68)	3 (7.14)	254 (26.82)	257 (25.99)			
65–69	13 (12.75)	576 (20.43)	589 (20.16)	6 (14.29)	202 (21.33)	208 (21.03)			
70–74	26 (25.49)	437 (15.5)	463 (15.85)	12 (28.57)	135 (14.26)	147 (14.86)			
≥75	52 (50.98)	442 (15.67)	494 (16.91)	19 (45.24)	66 (6.97)	85 (8.59)			
School education (*n*, %)							0.01	<0.001	<0.001
<6 y	96 (94.12)	1948 (69.1)	2044 (69.98)	33 (78.57)	454 (47.94)	487 (49.24)			
≥6 y	6 (5.88)	871 (30.9)	877 (30.02)	9 (21.43)	493 (52.06)	502 (50.76)			
Marriage (*n*, %)							0.045	<0.001	<0.001
Spouse-absent	60 (58.82)	811 (28.77)	871 (29.82)	17 (40.48)	129 (13.62)	146 (14.76)			
Married	42 (41.18)	2008 (71.23)	2050 (70.18)	25 (59.52)	818 (86.38)	843 (85.24)			
Hypertension (*n*, %)							<0.001	0.02	<0.001
Yes	49 (48.04)	726 (25.75)	775 (26.53)	35 (83.33)	280 (29.57)	315 (31.85)			
No	53 (51.96)	2093 (74.25)	2,146 (73.47)	7 (16.67)	667 (70.43)	674 (68.15)			
Cardiovascular Disease (*n*, %)^b^							0.04	0.47	0.292
Yes	2 (1.96)	155 (5.5)	157 (5.37)	4 (9.52)	58 (6.12)	62 (6.27)			
No	100 (98.04)	2,664 (94.5)	2,764 (94.63)	38 (90.48)	889 (93.88)	927 (93.73)			
Stroke (*n*, %)							<0.001	0.23	0.53
Yes	44 (43.14)	326 (11.56)	370 (12.67)	6 (14.29)	121 (12.78)	127 (12.84)			
No	58 (56.86)	2,493 (88.44)	2,551 (87.33)	36 (85.71)	826 (87.22)	862 (87.16)			
C-MMSE score (p25,p75)	13.57 (10.17)	22 (24.28)	25.34 (23.28)	15.12 (13.17)	24.9 (23.28)	25 (23.28)	<0.001	0.198	0.23

Note: Binary variables (age stratification, gender, education, marriage status, and comorbidities) are reported as *N* (%) unless otherwise noted. The proportion of different characteristics in each of the two groups is shown as a percentage with parentheses.

^a^ The *P*-value displays the outcome of the Student’s *t*-test conducted on two groups. P1 represents the comparison between all groups, P2 pertains to the comparison between patients with dementia, and P3 pertains to the comparison among participants without dementia.

^b^ Cardiovascular disease includes diseases such as coronary atherosclerotic heart disease, rheumatic heart disease, hypertensive heart disease, and pulmonary heart disease.

In the XRRCC1, 636 participants were lost to follow-up, 628 of whom did not have dementia, while 8 had dementia. In the XRRCC2, 155 were lost to follow-up, all of whom did not have dementia. The total participation rates were 78% for the XRRCC1 and 84% for the XRRCC2. There were no significant differences among the demographic characteristics of the participants with missing data and those who completed follow-up assessments in both the XRRCC1 and XRRCC2 (Supplementary Datas S1, S2). The population demographics were consistent with those of the Xi’an rural population at the beginning of the investigation in both cohorts, as shown in Supplementary Data S3.

### Prevalence of Dementia in the Two Cohorts

The XRRCC1 had 102 patients with dementia, while the XRRCC2 had 42 patients with dementia at baseline. The rates of crude prevalence of dementia in participants older than 55 years were 3.49% in the XRRCC1 and 4.25% in the XRRCC2. The age–sex standardized prevalence of dementia was 2.53% in the XRRCC1 and 4.45% in the XRRCC2. The prevalence of dementia was significantly higher in the XRRCC2 than it was in the XRRCC1 (OR = 1.79, 95%CI: 1.2–2.65, *P* = 0.004). Women had a significantly higher prevalence of dementia in the XRRCC2 than those in the XRRCC1 after adjusting for age, sex, and education (OR = 1.90, 95%CI: 1.13–3.22, *P* = 0.016) (Table 2).

**TABLE 2:** Prevalence and mortality of dementia in two cohorts (Xi’an rural district, China. 2020).

	XRRCC1			XRRCC2			Trends on rate between two cohorts (OR/HR)^a^	*P*-value of trend
	No.	Crude rate	Standardized rate^§^	No.	Crude rate	Standardized rate		
Dementia Prevalence								
Total	102	3.49 (2.88–4.24)	2.53 (2.08–3.07)	42	4.25 (3.14–5.75)	4.45 (3.85–5.14)	1.79 (1.20–2.65)	0.004*
Men	44	3.41 (2.64–4.41)	3.19 (2.55–4)	17	4.00 (2.49–6.43)	4.16 (3.31–5.21)	1.67 (0.90–3.07)	0.100
Women	58	3.59 (2.68–4.83)	3.38 (2.61–4.38)	25	4.43 (2.99–6.56)	4.85 (4.03–6.19)	1.90 (1.13–3.22)	0.016*
Mortality Dementia patients								
Total	63	62 (48.3–79.1)	35.47 (15.4–55.51)	15	35.7 (21.5–59.2)	13.05 (10.65–15.89)	0.33 (0.17–0.65)	0.01
Men	31	70.5 (49.5–98.51)	16.2 (2.35–30.04)	8	47.1 (23.5–94.1)	15.92 (11.87–21)	0.56 (0.22–1.47)	0.24
Women	32	55.17 (39–78.02)	19.28 (4.79–33.76)	7	28 (13.35–58.73)	10.2 (7.61–13.54)	0.15 (0.1–0.46)	0.001
Total population								
Total	334	11.43 (10.27–2.73)	10.44 (9.15–11.74)	57	5.76 (4.44–7.47)	2.31 (2.1–2.58)	0.34 (0.25–0.47)	<0.001
Men	177	14.47 (12.49–16.77)	5.86 (4.8–6.91)	36	8.47 (6.11–11.74)	3.29 (2.86–3.79)	0.67 (0.43–1.06)	0.08
Women	157	9.25 (7.9–10.81)	4.59 (3.83–5.34)	21	3.72 (2.43–5.71)	1.43 (1.19–1.73)	0.19 (0.12–0.32)	<0.001

Note: Rate and 95% confidence intervals (in parentheses) are provided.

Prevalence and mortality were shown as per 100 population.

^a^ Trends on the prevalence of dementia between the two cohorts are displayed with odds ratios (OR) for prevalence and hazards ratios (HR) for mortality.

P < 0.05 is considered a statistically significant difference and labeled with an asterisk.

**FIGURE 2:**
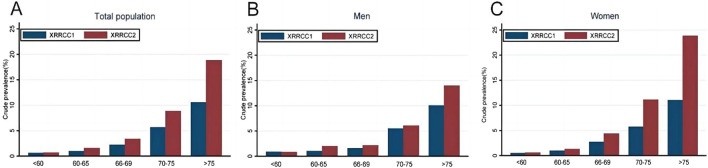
Prevalence of dementia in two cohorts based on age stratification (Xi’an rural district, China. 2020). **(A)** Comparison of dementia prevalence by age group in total population. **(B)** Comparison of dementia prevalence by age group among males. **(C)** Comparison of dementia prevalence by age group among females.

The prevalence of dementia continuously increased with age in both cohorts. The XRRCC2 had a significantly higher prevalence of dementia than that of the XRRCC1 for participants with >75 years of age. A significantly higher virtually identical trend was also observed for women ≥70 years of age and men ≥75 years of age (*P* < 0.05 for all age stratification) (Figure 2) (Supplementary Data S4).

### Mortality of Patients With Dementia During the Follow-Up Period in the Two Cohorts

#### Mortality of Patients With Dementia During the Follow-Up

At the end of the follow-up, 63 deaths were reported in the XRRCC1 and 15 deaths in the XRRCC2 among all patients with dementia. The patients with dementia in the XRRCC2 had significantly lower mortality than those in the XRRCC1 (35.7% vs. 62%; HR = 0.33, 95%CI: 0.17–0.65, *P* = 0.01) (Table 2). In addition, the age–sex standardized mortality was significantly lower in the XRRCC2 than it was in the XRRCC1 (13.05% vs. 35.47%). A significant reduction in mortality was observed among women (HR = 0.15, 95%CI: 0.1–0.46, *P* = 0.001) with dementia. The mortality of the total population was significantly lower in the XRRCC2 than it was in the XRRCC1 (HR = 0.34, 95%CI: 0.25–0.47, *P* < 0.001). This decreased mortality of the total population was most significant among women (HR = 0.19, 95%CI: 0.12–0.32, *P* < 0.001). Supplementary Data S5 shows the age distribution of the deceased patients with dementia for both cohorts.

**FIGURE 3:**
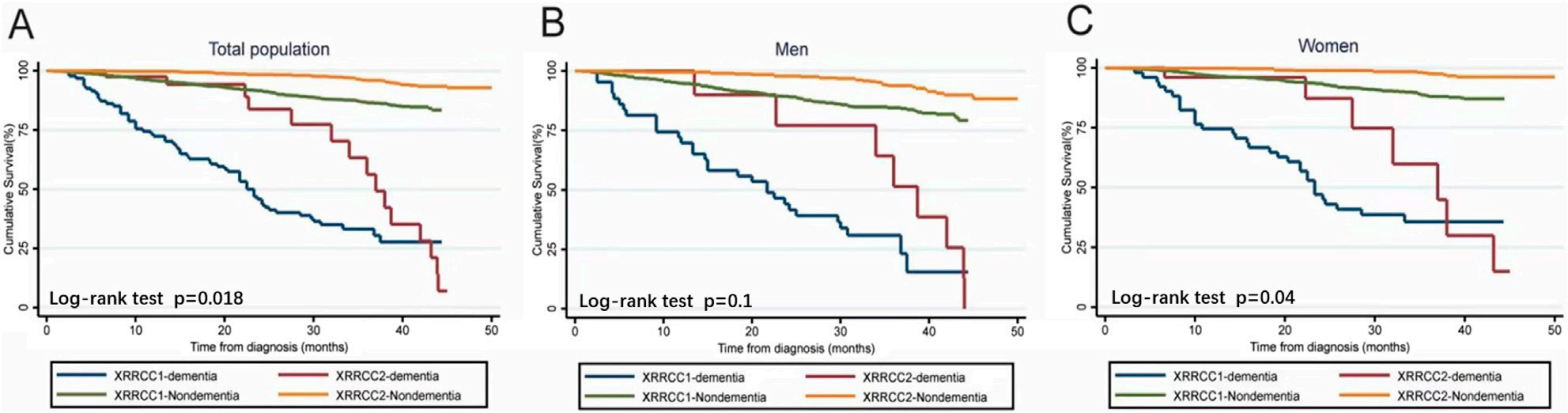
Survival status of people with dementia and total population in two cohorts (Xi’an rural district, China. 2020). **(A)** Comparison of Kaplan-Meier survival curves for two cohorts within the total population. **(B)** Comparison of Kaplan-Meier survival curves for two cohorts among males. **(C)** Comparison of Kaplan-Meier survival curves for two cohorts among females.

The average life expectancy was 1.6 years in the XRRCC1 and 3.2 years in the XRRCC2 after the diagnosis of dementia. As shown in the Kaplan–Meier survival curves, the mortality of the patients with dementia was significantly higher than that of the patients without dementia in both the XRRCC1 and XRRCC2. In addition, the life expectancy of the XRRCC2 members was significantly longer than that of the XRRCC1 members, irrespective of whether they had dementia or not (Log-rank *P* < 0.001) (Figure 3) (Supplementary Data S6). The XRRCC2 had a significantly lower mortality rate than that of the XRRCC1.

#### Factors Associated With Temporal Trends Related to the Development of Dementia

**FIGURE 4:**
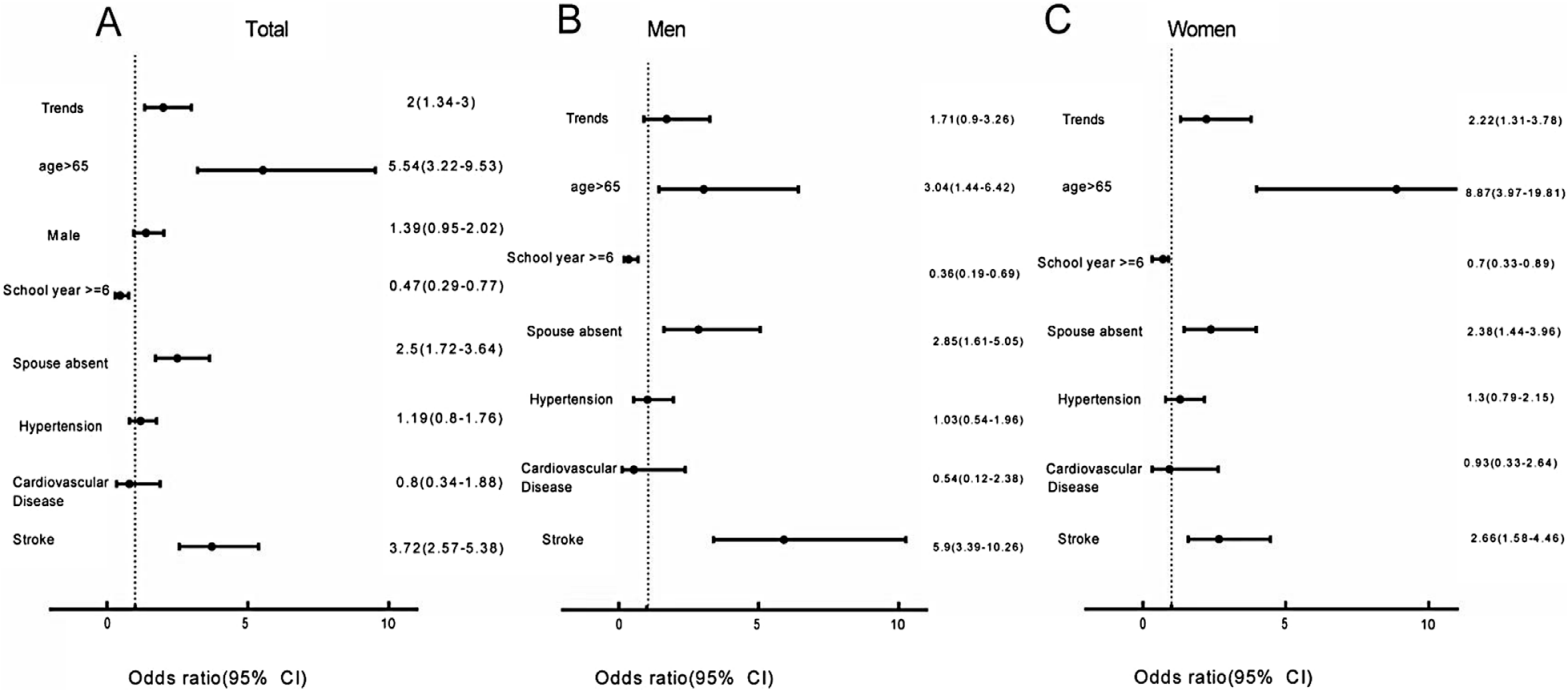
Factors contributing to change in the trends of dementia prevalence (Xi’an rural district, China. 2020). **(A)** Factors affecting changing trends in dementia prevalence in the total population. **(B)** Factors affecting changing trends in dementia prevalence among males. **(C)** Factors affecting changing trends in dementia prevalence among females.

Multivariate logistic regression analysis of the pooled data showed that the increase in the prevalence of dementia is associated with age (>65 years) (OR = 5.54, 95%CI: 3.22–9.53), spouse-absent status (OR = 2.5, 95%CI: 1.72–3.64), and stroke (OR = 3.72, 95%CI: 2.57–5.38). Completion of more than 6 years of school-age education is negatively associated with the prevalence of dementia (OR = 0.47, 95%CI: 0.29–0.77) (Figure 4). The sensitivity analysis results remained unchanged after implementing different variables. The missing values, when assigned under both the worst-case and best-case scenarios, did not affect the conclusions. We report the results obtained under different scenarios in Supplementary Data S7. The conclusions drawn from the competing risks model remained consistent even when different competing risks were substituted (Supplementary Datas S8).

## Discussion

By pooling data from two cohorts belonging to the same districts over 17 years, we found that the prevalence of dementia increased, the dementia-associated mortality decreased, and the life expectancy of patients with dementia increased. Patients older than 65 years, without a spouse, and with a stroke history had a higher prevalence of dementia. Conversely, those with more than 6 years of school-age education had a lower prevalence.

Although more than 100 epidemiological studies on dementia have been conducted in China [11], the secular trends on the prevalence of dementia reported so far remain inconsistent. Most studies on the trends of the prevalence of dementia in China comprise meta-analyses, system reviews, or history comparisons rather than any longitudinal cohort study. Variations in factors such as sampling geographical areas, diagnostic criteria, age of the surveyed population, and comparison of different cohorts may impact the validity and reliability of the secular trends of the prevalence of dementia in China.

The present study compared two cohorts from the same region using the same investigational procedures and diagnostic criteria at two different time periods. The crude analysis and age–sex standardized analysis results suggested that the prevalence of dementia was significantly higher in the XRRCC2 than it was in the XRRCC1. This prevalence significantly increased over the study period (17 years). The prevalence depends on the incidence and survival time. We also performed a cross-sectional study, in which we did not investigate the incidence of dementia. We found that although the mortality of patients with dementia decreased, their life expectancy increased. Hence, it can be suggested that an extended survival period is a significant risk factor for the increase in the prevalence of dementia in China. Over the past 20 years, rapid economic development, improved lifestyles, and increased investment in healthcare have helped increase the life expectancy of the older population of China. Advanced medical technologies, new medications and treatments, and improved diagnostic tools and screening methods have helped reduce the mortality rate of people with chronic diseases such as hypertension, diabetes, and cardiovascular disease [19], thereby substantially prolonging the life expectancy of the ageing population in China. In addition, we found that the difference in the increase in prevalence and the decrease in mortality was most significant among women. The difference between men and women could be explained by the observed small sample size, as 95%CI of the prevalence rates for men and women almost overlapped. Additionally, the improvement in medical care has significantly increased the average lifespan of women, surpassing that of men. As a result, women may become more susceptible to the development of these age-related neurological conditions compared to men.

Adults older than 65 years, without a spouse, and with a stroke history were positively associated with an increased prevalence of dementia, while those with more than 6 years of school-age education were negatively associated with dementia. The prevalence of dementia increased with age in both cohorts. The XRRCC2 cohort showed a more prominent, sharp upward trend for the prevalence of dementia. This prevalence almost doubled every 5 years, consistent with that reported in previous studies [20, 21]. Because the mean age of both cohorts was identical and ageing had the most significant odds among all factors in the regression models, we speculate that demographic changes due to ageing, particularly the increase in the 65-year-old population, are the primary reasons for the rising prevalence of dementia in rural China.

The prevalence of hypertension and stroke significantly increased in the XRRCC2 than in the XRRCC1. The pooling analysis showed that stroke is a significant risk factor for dementia. Medical care and treatment advancements have helped increase the survival period of patients with severe strokes, except those with vascular dementia resulting from stroke. However, patients with severe strokes are more likely to develop dementia. Hypertension, particularly midlife high blood pressure, is correlated with a higher risk for late-life cognitive impairment [22] and Alzheimer’s disease [23]. Emerging evidence has shown that the deleterious effect of high blood pressure on cognition occurs across one’s lifespan, increasing the risk for both early-onset and late-life dementia [24, 25]. Prolonged exposure to chronic hypertension has been found to change the transport of amyloid-beta peptides in plasma [26]. These changes are associated with the development of dementia. Because of poor access to healthcare services, people living in rural areas face severe challenges in receiving timely medical diagnoses and interventions, resulting in a high incidence of hypertension [27] and poor adherence to hypertension control [28], ultimately leading to prolonged exposure to hypertension [7]. In our study, we investigated only the history of hypertension. We did not study the responsiveness of rural residents to hypertension medication interventions. Further research on patient reactions to hypertension therapy may be necessary to fully understand the causes of this trend.

Patients with more than 6 years of school-age education were significantly higher in the XRRCC2 than in the XRRCC1. These individuals exhibited a negative correlation with the prevalence of dementia. This result is consistent with that reported in previous studies on the prevalence of dementia in developed urban areas [29]. Hence, it can be suggested that school-age education has a protective effect on dementia. This could be because sufficient early school-age education can create a cognitive reserve, which may help the brain tolerate pathological processes and prevent cognitive decline in later years [30]. Individuals with lower levels of education may need more awareness about the importance of a healthy lifestyle. They may also have difficulty comprehending medical instructions, which can adversely impact their adherence to the treatment of chronic diseases and result in poorer control of risk factors [31]. The Framingham Heart Study found that the decrease in the prevalence of dementia and related mortality in the later cohort was primarily due to improved cardiovascular health over time in individuals with education levels at least above high school level [32]. Besides, having a more extended education during childhood often results in higher economic status. Such individuals can overcome the inequity between rural and urban areas and have more access to high-quality medical support. The risk of developing dementia is estimated to decrease by approximately 11% for each additional school-age year of education [33]. According to the Alzheimer’s Association, increasing access to education can decrease the prevalence of dementia by 6.2 million cases by 2050 worldwide [34]. The current emphasis on promoting compulsory education has improved the education level of the rural residents of China. We believe this positive trend could help reduce the prevalence of dementia in rural China.

The number of individuals without a spouse significantly decreased in rural China during the study period. In addition, we found that the marriage status impacts the temporal trend regarding the prevalence of dementia. A stable and satisfying marriage is positively associated with a lower risk of dementia after retirement, possibly because of the ease of maintaining a healthy lifestyle and increased social stimulation, which helps combat loneliness in old age. However, the extended life expectancy and spouse-absent status imply social isolation and loneliness, an unhealthy lifestyle, and increased levels of stress or depression, all of which are associated with a higher risk of developing dementia [35, 36].

Compared to nursing homes, Chinese older adults are more inclined to choose home care as a form of elderly care, as it can provide more convenience and autonomy. It also allows them to maintain social connections with their families and communities. In contrast, elderly empty nesters are more inclined to opt for nursing care when they encounter challenges caring for themselves. Urban–rural social and economic inequalities may result in poor healthcare services for rural residents. This situation worsens the prognosis of spouse-absent patients. We recommend improving social support, particularly for those older adults who are single, divorced, or widowed, to prevent an increase in the burden of dementia in rural China.

Women with dementia reported an increased prevalence but decreased mortality, confirming the sex differences in dementia risk factors, including the APOE gene, stroke, and hypertension [37]. However, we did not find gender disparities in the risk factors associated with dementia at the baseline investigation. Although men with stroke are more likely to experience an increase in the temporal trends of dementia compared to women in pooled analysis, the difference in this risk factor is insufficient to fully explain the gender disparity in dementia-related temporal trends. We speculate that the gender difference could be mainly attributed to the extended lifespan of patients with dementia. Our regression analysis demonstrated that age is another risk factor with a greater impact on women than on men, increasing the temporal trends for the prevalence of dementia. Medical advancements in rural China have reduced mortality rates [38], ultimately shifting the spectrum of fatal diseases. Compared to women with dementia, men with dementia may exhibit poorer management of risk factors such as high blood pressure, social isolation, and comorbidities [39, 40], which contribute to their high mortality. Further studies investigating the cause of death in rural dementia patients must verify this hypothesis.

This is the first study to provide direct evidence regarding temporal trends in the prevalence of dementia, mortality, and survival states of the rural population of China. However, the study has several limitations as well. First, this study compared two small population-based cohorts over a short follow-up period. The short observation periods and discontinuous data collection limited our ability to use jointpoint regression for calculating annual trends in dementia incidence [41]. Although we adjusted for confounding factors and used stratification for the analysis, the random error and bias in the study limited the generalizability of the findings to the larger population. The small study sample also prevented further analysis of the various subtypes of dementia (e.g., Alzheimer’s disease and vascular dementia). Therefore, future studies with larger sample sizes and longer follow-up times are necessary. Second, in many cases, the exact cause of death was not registered, which limited further discussion on mortality rates only to dementia rather than mortality rates specific to certain diseases in patients with dementia. It is necessary to register the exact cause of death and discuss its potential effect on the competing risk of dying from dementia or other reasons such as tumor [42] to better explain the increased life expectancy and an accurate estimation of dementia risk [43]. Third, the prevalence of dementia dramatically increases with age, even in those over the age of 90 years [44]. We need to conduct more investigations on remarkably aged people to accurately describe temporal changes regarding the prevalence of dementia among the older rural population of China.

Numerous drug therapies are currently being investigated for their potential impact on the incidence of dementia. These include treatments aimed at reducing amyloid-beta peptides [45] that form plaques in the brain or drugs designed to reduce neuroinflammation associated with the progression of dementia [46]. Future research may focus on exploring the combination of drug therapies with lifestyle modifications for a more comprehensive approach to dementia prevention.

In conclusion, by comparing the prevalence of dementia in two cohorts in rural China, we demonstrated that the prevalence of dementia significantly increased over 17 years, while mortality significantly decreased. Ageing, spouse-absent status, and stroke may increase the prevalence of dementia, while a higher school-age education level may partially slow down this increase. Control of hypertension and stroke, a higher school-age education level, and social support for older adults are essential for preventing dementia. A more extensive and comprehensive longitudinal cohort study should be conducted to examine the evolving trends regarding the prevalence of dementia and the effectiveness of prevention strategies.
