# Supplementary material for: Trends on Prevalence, All-Cause Mortality, and Survival Status of Dementia Patients in Rural China Based on Pooling Analysis
Source: Int J Public Health. 2024 Aug 22;69:1606786. doi: 10.3389/ijph.2024.1606786 (PMC11374651; doi:10.3389/ijph.2024.1606786)
Supplement: Supplementary file 1 [file DataSheet1.docx]

**Supplemental data 1**. Data missing at baseline (Xi’an rural district, China. 2020)

| Variables | XRRCC1 | | |  | XRRCC2 | | |
| --- | --- | --- | --- | --- | --- | --- | --- |
|  | Total | Men | Women |  | Total | Men | Women |
| Marriage status (*n*) |  |  |  |  |  |  |  |
| Available | 2837 | 1637 | 1200 |  | 973 | 417 | 556 |
| Missing data | 85 | 62 | 23 |  | 16 | 8 | 8 |
| Hypertension (*n*) |  |  |  |  |  |  |  |
| Available | 2895 | 1684 | 1211 |  | 984 | 422 | 562 |
| Missing data | 27 | 15 | 12 |  | 5 | 3 | 2 |
| Cardiovascular disease (*n*) |  |  |  |  |  |  |  |
| Available | 2909 | 1693 | 1216 |  | 984 | 422 | 562 |
| Missing data | 13 | 6 | 7 |  | 5 | 3 | 2 |
| Stroke (*n*) |  |  |  |  |  |  |  |
| Available | 2909 | 1693 | 1216 |  | 984 | 422 | 562 |
| Missing data | 13 | 6 | 7 |  | 5 | 3 | 2 |

**Supplemental data 2.** Overall available and lost to follow-up cases of XRRCC1 and XRRCC2 cohorts (Xi’an rural district, China. 2020)

| Variables | XRRCC1 | |  | XRRCC2 | |
| --- | --- | --- | --- | --- | --- |
|  | Available | Lost to Follow-up |  | Available | Lost to Follow-up |
| Age (*n*) |  |  |  |  |  |
| 55–59 y | 504 | 150 |  | 253 | 39 |
| 60–64 y | 577 | 144 |  | 221 | 36 |
| 65–69 y | 464 | 125 |  | 177 | 31 |
| 70–74 y | 349 | 114 |  | 113 | 34 |
| 75–79 y | 241 | 75 |  | 39 | 8 |
| ≥80 y | 150 | 28 |  | 29 | 7 |
| Sum | 2285 | 636 |  | 832 | 155 |

Note: Participation rates are 78% in XRRCC1 and 84% in XRRCC2.

**Supplemental data 3.** Age and gender distribution of participants and total population of Xi’an rural districts (Xi’an rural district, China. 2020)

| Demographic Characteristics | Epidemiology Cohorts | |  | Total Population of Xi’an Rural Districts^†^ | |
| --- | --- | --- | --- | --- | --- |
|  | XRRCC1 | XRRCC2 |  | 1997 | 2014 |
| Sex (*n*, %) |  |  |  |  |  |
| Men | 1.2(42) | 0.4(43) |  | 148(49) | 195(48) |
| Women | 1.7(58) | 0.5(57) |  | 154(51) | 212(52) |
| Age (*n*, %) |  |  |  |  |  |
| 55–64 y | 123(51) | 123(56) |  | 169(56) | 232(57) |
| 65–74 y | 123(33) | 123(56) |  | 97(32) | 138(34) |
| ≥75 y | 123(16) | 123(56) |  | 36(12) | 37(9) |

Note: Demographic data of the Xi’an population were collected from the website of the statistics bureau for XRRCC1 in 1997 and XRRCC2 in 2014 (http://tjj.xa.gov.cn/tjnj/2014/tjnj/indexch.htm).**Supplemental data 4.** Age stratification of people with dementia and a comparison of the two cohorts (Xi’an rural district, China. 2020)

|  | | XRRCC1 | | |  | XRRCC2 | | | *P*-value |
| --- | --- | --- | --- | --- | --- | --- | --- | --- | --- |
|  |  | Total  (*n*) | Dementia  (*n*) | Prevalence  (%) |  | Total(*n*) | Dementia(*n*) | Prevalence  (%) |  |
| Total |  |  |  |  |  |  |  |  |  |
| 55–59 y | | 654 | 4 | 0.61 |  | 292 | 2 | 0.68 | 0.59 |
| 60–64 y | | 721 | 7 | 0.97 |  | 257 | 4 | 1.56 | 0.33 |
| 65–69 y | | 589 | 13 | 2.21 |  | 208 | 7 | 3.37 | 0.26 |
| 70–74 y | | 463 | 26 | 5.62 |  | 147 | 13 | 8.84 | 0.14 |
| ≥75 y | | 494 | 52 | 10.53 |  | 85 | 16 | 18.82 | 0.04* |
| Men |  |  |  |  |  |  |  |  |  |
| 55–59 y | | 238 | 2 | 0.84 |  | 122 | 1 | 0.82 | 0.73 |
| 60–64 y | | 297 | 3 | 1.01 |  | 101 | 2 | 1.98 | 0.38 |
| 65–69 y | | 256 | 4 | 1.56 |  | 93 | 2 | 2.15 | 0.51 |
| 70–74 y | | 183 | 10 | 5.46 |  | 66 | 4 | 6.06 | 0.54 |
| ≥75 y | | 249 | 25 | 10.04 |  | 43 | 6 | 13.95 | 0.32 |
| Women |  |  |  |  |  |  |  |  |  |
| 55–59 y | | 416 | 2 | 0.48 |  | 170 | 1 | 0.59 | 0.64 |
| 60–64 y | | 424 | 4 | 0.94 |  | 156 | 2 | 1.28 | 0.51 |
| 65–69 y | | 333 | 9 | 2.7 |  | 115 | 5 | 4.35 | 0.29 |
| 70–74 y | | 280 | 16 | 5.71 |  | 81 | 9 | 11.11 | 0.04* |
| ≥75 y | | 245 | 27 | 11.02 |  | 42 | 10 | 23.81 | 0.03* |

Note: Differences between the XRRCC1 and XRRCC2 are compared using a chi-square test; those with statistically significant differences are marked with an asterisk.

**Supplemental data 5.** Age stratification of patients with dementia dying in XRRCC1 and XRRCC2 cohorts (Xi’an rural district, China. 2020)

| Demographic Characteristics | | XRRCC1 | |  | XRRCC2 | |
| --- | --- | --- | --- | --- | --- | --- |
|  |  | Total Deaths  (*n*) | Dementia  (*n*) |  | Total Deaths  (*n*) | Dementia  (*n*) |
| Total |  |  |  |  |  |  |
| 55–59 y | | 34 | 2 |  | 5 | 0 |
| 60–64 y | | 44 | 2 |  | 8 | 0 |
| 65–69 y | | 61 | 12 |  | 13 | 2 |
| 70–74 y | | 69 | 11 |  | 7 | 2 |
| ≥75 y | | 126 | 36 |  | 24 | 11 |
| Men |  |  |  |  |  |  |
| 55–59 y | | 14 | 1 |  | 4 | 0 |
| 60–64 y | | 25 | 0 |  | 5 | 0 |
| 65–69 y | | 28 | 6 |  | 7 | 0 |
| 70–74 y | | 42 | 5 |  | 4 | 1 |
| ≥75 y | | 68 | 19 |  | 16 | 7 |
| Women |  |  |  |  |  |  |
| 55–59 y | | 20 | 1 |  | 1 | 0 |
| 60–64 y | | 19 | 2 |  | 3 | 0 |
| 65–69 y | | 33 | 6 |  | 6 | 2 |
| 70–74 y | | 27 | 6 |  | 3 | 1 |
| ≥75 y | | 58 | 17 |  | 8 | 4 |

**Supplemental data 6.** Comparison of the survival status of XRRCC1 and XRRCC2 cohorts using a log-rank test (Xi’an rural district, China. 2020)

|  |  | χ2 Test | *P*-value |
| --- | --- | --- | --- |
| Total |  | 63.89 | <0.001* |
|  | Dementia | 5.6 | 0.018* |
|  | No dementia | 57.55 | <0.001* |
| Men |  | 27.18 | <0.001* |
|  | Dementia | 2.68 | 0.1 |
|  | No dementia | 24.35 | <0.001* |
| Women |  | 37.1 | <0.001* |
|  | Dementia | 3.33 | 0.04* |
|  | No dementia | 33.96 | <0.001* |

Note: Differences in the survival status of XRRCC1 and XRRCC2 (overall and subgroups) are compared using a log-rank test. Statistically significant differences are marked with an asterisk.

**Supplemental data 7.** Logistic regression on factors attributed to changes in dementia prevalence with pooled data after multiple imputations of pooling data (Xi’an rural district, China. 2020)

|  | Estimation of Scenario 1 | |  | Estimation of Scenario 2 | |
| --- | --- | --- | --- | --- | --- |
|  | OR | *P*-value |  | OR | *P*-value |
| Total |  |  |  |  |  |
| Age >65 y | 3.37(1.65–4.12) | <0.001* |  | 1.79(1.2–2.65) | 0.004* |
| Men | 0.8(0.27–1.58) | 0.68 |  | 1.67(0.9–3.07) | 0.1 |
| School year education ≥6 | 0.26(0.18–0.39) | <0.001* |  | 0.45(0.16–0.67) | 0.016* |
| Spouse-absent | 2.44(1.76–3.37) | <0.001* |  | 2.04(1.49–2.82) | 0.006* |
| Hypertension | 1.01(0.74–1.37) | 0.953 |  | 0.96(0.62–1.47) | 0.74 |
| Cardiovascular disease | 1.29(0.97–1.7) | 0.076 |  | 2.68(0.15–6.04) | 0.7 |
| Stroke | 5.02(3.23–7.82) | <0.001* |  | 4.56(2.93–7.09) | <0.001* |
| Men |  |  |  |  |  |
| Age >65 | 6.5(2.76–15.31) | <0.001* |  | 2.6(1.4–3.1) | <0.001* |
| School year education ≥6 | 0.34(0.16–0.69) | 0.003* |  | 0.32(0.16–0.67) | 0.002* |
| Spouse-absent | 1.51(0.24–1.81) | 0.088 |  | 1.67(0.9–1.13) | 0.1 |
| Hypertension | 0.91(0.37–2.2) | 0.829 |  | 1.12(0.45–2.78) | 0.812 |
| Cardiovascular disease | 2.33(0.73–7.39) | 0.151 |  | 3.91(1.12–13.63) | 0.032 |
| Stroke | 6.32(2.9–13.76) | <0.001* |  | 5.74(2.6–12.66) | <0.001* |
| Women |  |  |  |  |  |
| Age >65 y | 2.63(1.46–3.85) | 0.003* |  | 1.07(0.46–3.39) | 0.59 |
| School year education ≥6 | 0.18(0.11–0.3) | <0.001* |  | 0.17(0.1–0.28) | 0.002* |
| Spouse-absent | 3.47(2.44–4.93) | <0.001* |  | 3.55(2.5–5.04) | 0.002* |
| Hypertension | 1.06(0.76–1.46) | 0.742 |  | 0.92(0.56–1.5) | 0.725 |
| Cardiovascular disease | 1.27(0.95–1.7) | 0.114 |  | 1.35(0.4–4.52) | 0.631 |
| Stroke | 5.33(3.05–9.31) | <0.001* |  | 5.52(3.15–9.69) | <0.001* |

Note: Statistically significant factors are marked with an asterisk.

**Supplemental data 8.** Comparison of the mortality of XRRCC1 and XRRCC2 cohorts with competing risk factor analysis (Xi’an rural district, China. 2020)

|  |  | HR | 95%CI | *P*-value |
| --- | --- | --- | --- | --- |
| Model 1 |  |  |  |  |
|  | Cohort | 0.35 | (0.26–0.47) | <0.001* |
|  | Dementia | 3.52 | (2.64–4.7) | <0.001* |
|  | Age | 1.09 | (1.08–1.1) | <0.001* |
|  | Gender | 1.65 | (1.33–2.06) | <0.001* |
|  | Education | 0.95 | (0.92–0.98) | <0.001* |
|  | hypertension | 1.02 | (0.77–1.33) | 0.91 |
|  | stroke | 1.74 | (1.34–2.27) | <0.001* |
| Model 2 |  |  |  |  |
|  | Cohort | 0.61 | (0.39–0.95) | 0.03 |
|  | dementia | 2.35 | (1.4–3.94) | <0.001* |
|  | age | 1.08 | (1.06–1.1) | <0.001* |
|  | gender | 1.97 | (1.35–2.88) | <0.001* |
|  | education | 1 | (0.95–1.05) | 0.92 |
|  | Cardiovascular Disease | 3.14 | (1.72–5.75) | <0.001* |
|  | stroke | 1.23 | (0.78–1.96) | 0.37 |
| Model 3 |  |  |  |  |
|  | Cohort | 0.51 | (0.38–0.7) | <0.001* |
|  | dementia | 2.4 | (1.75–3.31) | <0.001* |
|  | age | 1.1 | (1.09–1.12) | <0.001* |
|  | gender | 1.56 | (1.23–1.98) | <0.001* |
|  | education | 0.96 | (0.93–0.99) | 0.01 |
|  | Cardiovascular Disease | 1.85 | (1.07–3.2) | 0.03 |
|  | hypertension | 0.92 | (0.72–1.17) | 0.5 |

Note: Comparing the temporal trends of mortality rates between cohorts using competing risks regression. The competing risk is cardiovascular disease in Model 1, hypertension in Model 2, and stroke in Model 3.
